# A late presentation of benign cephalic histiocytosis

**DOI:** 10.1016/j.jdcr.2024.10.030

**Published:** 2024-11-23

**Authors:** Madison A. Hackley, Nicholas D. Brownstone, Shayan Waseh, Simo Huang, Jason B. Lee, Sylvia Hsu

**Affiliations:** a Department of Dermatology, Temple University Lewis Katz School of Medicine, Philadelphia, Pennsylvania; b Department of Dermatology and Cutaneous Biology, Sidney Kimmel Medical College at Thomas Jefferson University, Philadelphia, Pennsylvania

**Keywords:** BCH, benign cephalic histiocytosis, histiocytosis

## Case report

**Fig 1:**
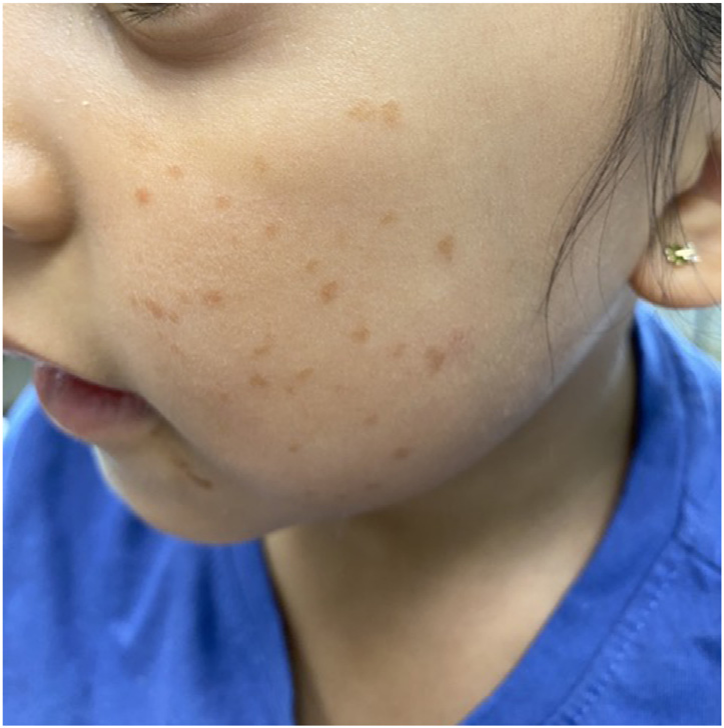
Multiple, *light-brown* pigmented macules and papules on the left cheek. Similar lesions were present on the right cheek.

**Fig 2:**
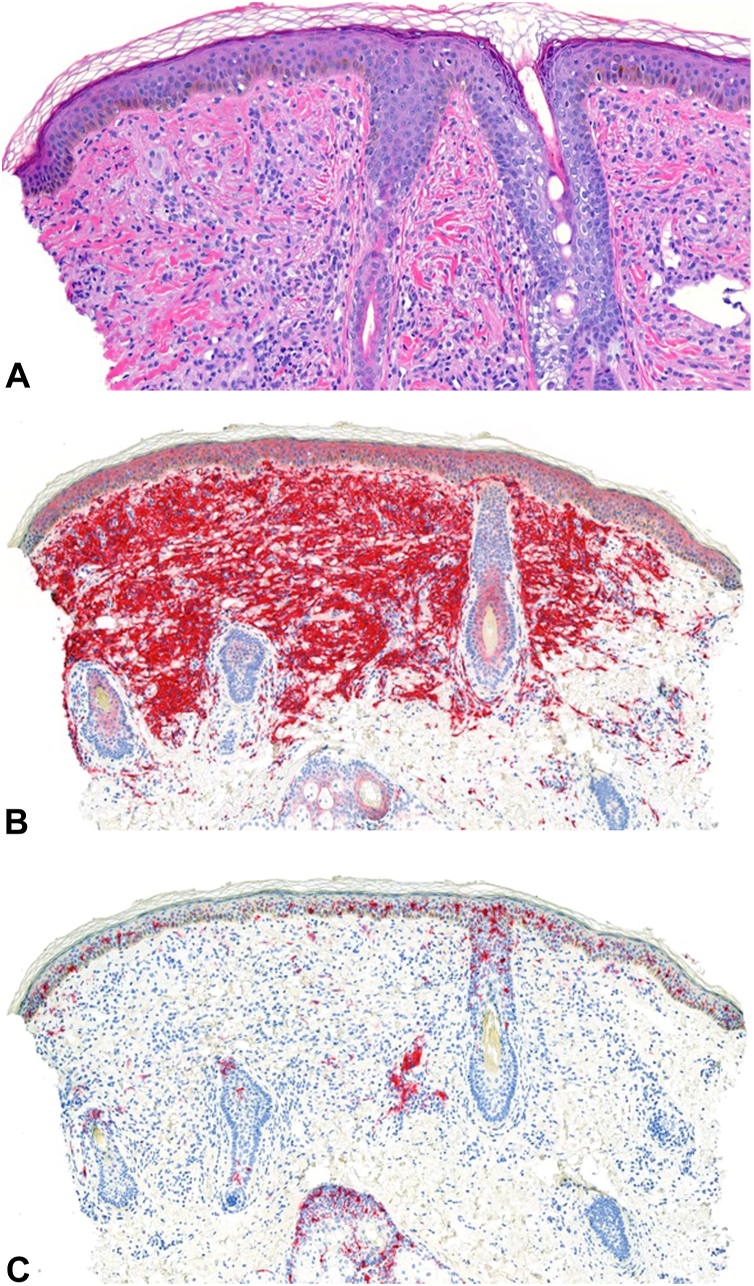
Benign cephalic histiocytosis. There are oval-to-round pale histiocytes, some of them with foamy cytoplasm, throughout the dermis accompanied by perivascular lymphocytes (**A,** H&E 200×). CD163 stain highlights a dense infiltrate of histiocytes within the superficial dermis (**B,** 100×). CD1a stain, a specific marker for Langerhans cells, fails to highlight the lesion (**C,** 100×).

A 5-year-old girl presented to the dermatology clinic with numerous hyperpigmented macules on the bilateral cheeks (Fig 1). According to the patient’s mother, the macules had spontaneously appeared 6 months prior to the patient’s presentation to the clinic. The macules were not painful or pruritic. The patient’s past medical history was noncontributory. On examination, there were multiple 1-mm, round, well-demarcated, light brown macules on the bilateral cheeks. A few lesions exhibited the morphology of papules. A punch biopsy was performed and demonstrated a prominent histiocytic infiltrate with foci of foamy cells in the superficial dermis (Fig 2, *A*), highlighted by CD163 (Fig 2, *B*) and CD68 stains. A CD1a stain failed to highlight the cells (Fig 2, *C*). The combination of these dermatopathology findings with the clinical presentation was consistent with a diagnosis of benign cephalic histiocytosis (BCH).

## Introduction

Benign cephalic histiocytosis (BCH) is a subtype of non-Langerhans cell histiocytosis that occurs in young children. BCH has an average age onset of 15 months, although a recently published case series found the average age of presentation to occur before 6 months of age.^1^^,^^2^ BCH typically presents as multiple small, asymmetric, and asymptomatic, yellow-brown macules, predominantly on the face and neck.^1^^,^^2^ The pathogenesis of BCH is still largely unknown.^3^ The diagnosis of BCH is usually clinical, although histopathology usually shows a histiocytic infiltrate in the superficial dermis. Immunohistochemical staining of BCH lesions is positive for factor XIIIa, CD163, and CD68, but negative for CD1a, langerin, and S100.^1^^,^^3^

## Discussion

Without confirmatory biopsy results, BCH may be easily misdiagnosed as other non-Langerhans cell subtypes.^3^ Both BCH and juvenile xanthogranuloma share similar immunohistologic markers: CD68, CD163, and Factor XIIIa.^3^ Other forms of non-Langerhans cell histiocytosis are less prone to spontaneous regression in comparison to BCH.^3^ Differentiating between the subtypes can help guide clinical decision-making about treatment.

The prognosis of BCH is very favorable. BCH is self-limiting, and the lesions spontaneously regress over a period of months to years.^1^^,^^2^ Some cases of BCH can be characterized by periods of exacerbation or escalation to generalized eruptive histiocytosis.^3^ Treatment of the lesions is not necessary. Recent literature has reported the successful treatment of BCH with rapamycin in a handful of pediatric cases.^4^^,^^5^ However, regression of lesions resulted in mild scarring in a small portion of reported BCH cases.^1^^,^^4^ Cosmetic correction of residual scarring can be considered on an individual basis.

Non-Langerhans cell histiocytosis subtypes, including BCH, have been associated with diseases such as diabetes insipidus and insulin-dependent diabetes mellitus.^6^^,^^7^ However, these associations are exceedingly rare, and there are no serious systemic complications or comorbidities linked to BCH.^8^ Therefore, no further workup was needed for our asymptomatic patient.

BCH is an exceedingly rare diagnosis, with only 60 cases reported in the literature.^2^ Differentiation between BCH and other non-Langerhans cell manifestations that are less prone to spontaneous regression, such as progressive mucinous familial histiocytosis and xanthoma disseminatum, is important.^3^ Additionally, it is crucial to recognize that BCH can manifest as late as the fifth or sixth year of life, as in our patient. Accurate diagnosis of BCH in pediatric populations requires the use of both clinical findings and immunohistological markers. This case report highlights an underrepresented, late presentation of this rare and underdiagnosed dermatologic entity.

## Conflicts of interest

None disclosed.
